# Prognostic Value of ICH Score and ICH-GS Score in Chinese Intracerebral Hemorrhage Patients: Analysis From the China National Stroke Registry (CNSR)

**DOI:** 10.1371/journal.pone.0077421

**Published:** 2013-10-16

**Authors:** Wenjuan Wang, Jingjing Lu, Chunxue Wang, Yilong Wang, Hao Li, Xingquan Zhao, on behalf of the investigators for the China National Stroke Registry (CNSR) Investigators

**Affiliations:** 1 Department of Neurology, Beijing Tiantan Hospital, Capital Medical University, Beijing, China; D'or Institute of Research and Education, Brazil

## Abstract

**Purpose:**

No strongevidenceofefficacycurrently exists for different intracerebral hemorrhage (ICH) scoring system in predicting the prognosis of ICH in the Chinese population. This study aimed to test the accuracyof the ICH score and the ICH grading scale (ICH-GS) score in predicting the favorable prognosis in a large cohort of ICH patients in China.

**Methods:**

This study was a multicenter, prospective cohort study. Patients diagnosed with ICH between September 2007 and August 2008 from the nationwide China National Stroke Registry (CNSR) databasewere screened andenrolled in this study. Demographics of the patients, treatments, mortalityas well as the clinic and radiologic findings of ICH were collected.AnICH score and anICH-GS score were evaluated for all the patients atadmission. Follow-ups were conducted by phone at 3, 6 and 12 months after ICH onset. The modified Rankin scale (mRS) score was used to evaluate favorable functional outcome and was obtained at hospital dischargeand duringthe 3-, 6- and 12-month follow-up visits.

**Results:**

There were 410 (12.6%) in-hospitalmortalityout of a total of 3,255 ICH patients. Thevalues of the Area Under Curve (AUC)at discharge, 3-, 6- and 12-month follow-up for ICH score were 0.72, 0.76, 0.76 and 0.75, respectively; whilethe numbers for the ICH-GS score were 0.71, 0.77, 0.78 and 0.78, respectively. At 6-month and 12-month follow-up, the ICH-GS score presented a significant better value in predicting favorable prognosis than did the ICH score (P=0.0003 and <0.0001, respectively).

**Conclusion:**

Both the ICH and ICH-GS scores were effective inaccurately predicting the favorable functional outcome of ICH in the Chinese population. For mid-term and long-term prediction, the ICH-GS score was superiorover the ICH score.

## Introduction

Intracerebral hemorrhage (ICH) accounts for 10-15% of all strokes in western countries and is one of the leading causes of death and disability worldwide[1–4]. Numerous results of studies on various aspects of the disease have been published. Multiple risk factors of the disease and the pathophysiology process of the secondary neurologic injuries of ICH have been well recognizedby the scientific community[4,5].However, physicians are still facing constant challenges in making treatment decisions forthe ICH patients. It is essential to develop a scale model that can accurately predict the prognosis of ICH based solely on the demographics of patients and the severity of the hemorrhage so that the physicians can use the scale to assess the impact of risk factors and the therapeutic efficacy of different treatment modalities[6]. Several scale model shave been developed and the ICH score and the ICH-GS score (Table S1) are among the most commonly used models to date.Both the ICHscore and the ICH-GS score have been well recognized in the Western world as valuable predictors of the prognosis of ICH. However, their performances in large Chinese populations have not been examined.

ICH accounts for 55% of all types of strokes in China[7].This percentage is much higher than that in the Western population[8–10]. There are also some regional differences within China. The incidence rate is higher in Northern China than in Southern China. Although cerebrovascular amyloidosis and oral anticoagulants play an important role in the occurrence of ICH in the Western world[4], they are less common in China. Chronic hypertension remains the dominant cause of ICH in China and other East Asian countries[11–13]. The distinctiveness of certain aspects in epidemiology, etiology and pathophysiology of ICH in the Chinese population may result in the different clinic characteristics and outcomes of ICH in China. Since no scale model has been developed to predict the functional outcome of ICH from the Chinese population, it is important to verify the prognostic value of existing models in Chinese ICH patients.

To assess whether ICH score or ICH-GS score can accurately predict short-term and long-term outcomes among the ICH patients in China, we selected ICH patients from the China National Stroke Registry (CNSR) and evaluated the efficacy of the models in predicting the prognosis, especially the favorable functional outcome, in the Chinese ICH patients.

## Methods

### Study design

This study was an observational, prospective cohort study. All the patients enrolled in the study were selected and screened from the CNSR database.

CNSR is a nationwide prospective registry of stroke patients, which was initiated in 2007 and was sponsored by the Chinese Ministry of Health. The registry has 132 participating hospitals from 27 provinces and 4 municipalities across China (including Hong Kong SAR). There are one hundred Grade III hospitals and 32 Grade II hospitals. These hospitals were strategically selected from the east, west and central regions of China to maximizeits geographic and demographic representativeness and to limit the variations among different regions in terms of the hospital size, infrastructure, population composition, et al[14]. 

### Patient eligibility

ICH was diagnosed according to World Health Organization (WHO) criteria[15]combined with brain computer tomography (CT) imaging. All patients were admitted during the one-year period from September 2007 to August 2008. The ICH patients who were ≥18 years old and were treated within 14 days after the ICH onset were eligible to be enrolled in the study. Outpatients, patients without hematoma measurements, primary intraventricular hemorrhage (IVH), ICH caused by tumor, disabilities prior to current ICH onset (with modified Rankin Scale[mRS]>2) and patients who did not consent to or were missing for follow-ups were excluded. 

This study was approved by the institutional review board at Beijing Tiantan Hospital. A written informed consent signed by the patient or his/her legal representative was obtained from all the patients enrolled in the study.

### Data Collection

ICH volume was determined using the ABC/2 method based on the initial CT scan[16,17].The ICH site and the presence of IVH in the initial CT scan were also documented.

The demographic data, e.g. age and gender, were also recorded. The information of the risk factors of ICH that collected in our study included history of ischemic/hemorrhagic stroke and history of current/previous antihypertensive agents, antiplatelet agents, or anticoagulants use.

Glasgow Coma Score(GCS), treatment during hospitalization, complications and medical status at discharge were also recorded.

### Follow-up and assessment of end points

Trained research personnel at follow-up center contacted the patients via telephone at 3, 6 and 12 months after the ICH onset. A standard questionnaire was used to obtain information of medication after discharge, current functional status and clinical outcomes. All follow-up data were recorded and archived in a central databank. A mRS score was calculated based on the information collected during each follow-up. Death was verified by death certificate issued by either the participating institution or the local resident registration agency. In rare cases, if the death certificate was not available, confirmations of death from two different sources (family members or local authorities) in two consecutive follow-ups were deemed admissible.

### Statistical methods

For descriptive analysis, proportions were used for categorical variable, mean with standard deviation (SD) was used for nominal variables. χ^2^ test was used to compare categorical variables; nonparametric Kruskal–Wallis one-way analysis of variance was used for nominal variables. We used logistic regression and the area under the curve (AUC) from the c-statistic to estimate the discrimination of the ICH score and the ICH-GS scores in predicting in-hospital, 3-month, 6-month and 12-month mortality and the favorable prognosis. Arandom bootstrap sample of 1000 cases was used to estimate the 95% confidence interval (95% CI) for each c-statistic. All tests were 2-tailed, and P<0.05 was considered statistically significant. All analyses were conducted using SAS ver.9.1(SAS Institute Inc.,Cary, North Carolina, USA).

## Results

**Figure 1:**
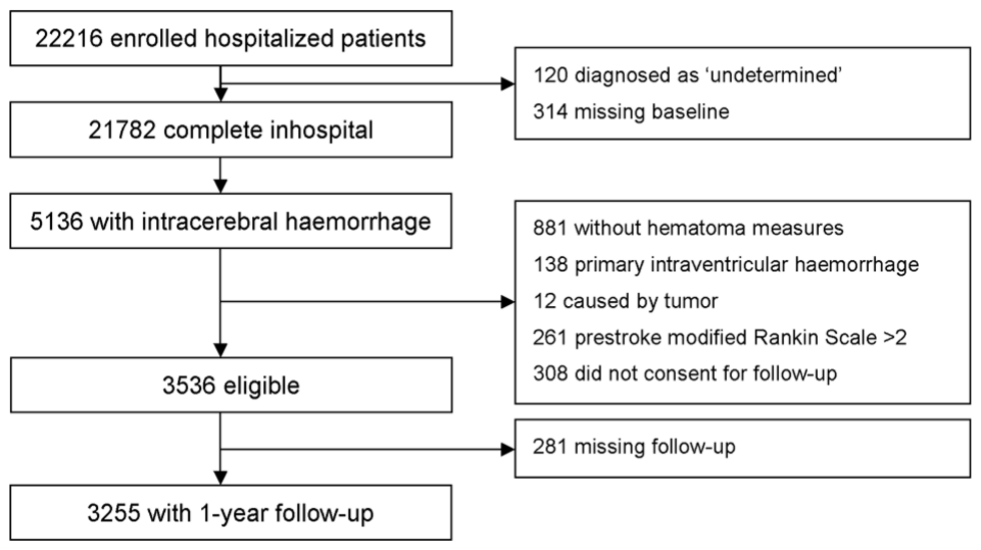
Flow diagram of the ICH patients screening process in this study.

Of all the 22,216 patients enrolled in CNSR, 5,136 were diagnosed with ICH and were screened for eligibility for this study. 1,881 of them were excluded because of: 1) 881 patients did not have the measurement of hematoma; 2) 138 patients had primary IVH; 3) 12 patients presented as intracranial tumor with hemorrhagic stroke; 4) 261 patients had disabilities prior to current incident of ICH; 5) 308 patients did not consent tothe follow-ups; 6) 281 patients lost follow-ups. The remaining 3,255 patients completed the 1-year follow-up and were included in the final statistical analysis (Figure 1).

In Table 1, we listed the characteristics of all the 3,255 ICH patients at baseline prior tothe initial treatment. The mean age of the ICH patients was 62.09± 13.09 years;1260 (38.7%)of them were women.13.6% of the patients had a history of previous ischemic stroke; 15.3% of them had a history of previous hemorrhagic stroke. 42.6% of the patients took antihypertensive medicine routinely. Only 8.9% and 1.0% of the patients took antiplatelet drugs and anticoagulants regularly. 12.1% of the patients had infratentorial hemorrhage; the mean hematoma volume was 23.00±30.35 ml for all ICH patients. In 962 (29.6%) cases, the hemorrhage ruptured into ventricle. Craniotomy was performed in 81 (2.7%) patients in this group.

**Table 1:** Baseline characteristics in intracerebral hemorrhage cohort.

Characteristics	Overall
Age, mean±SD	62.09 ± 13.09
Female, n(%)	1,260 (38.7%)
History of ischemic stroke, n(%)	443 (13.6%)
History of hemorrhagic stroke, n(%)	499 (15.3%)
Admission within 48 hours after onset, n(%)	2,823 (86.7%)
Antihypertensive agents, n(%)	1,388 (42.6%)
Antiplatelet agents, n(%)	291 (8.9%)
Anticoagulants, n(%)	32 (1.0%)
GCS score at admission, mean±SD	11.81 ±4.10
Hematoma site (Infratentorial), n(%)	393 (12.1%)
Hematoma volume, mean±SD	23.00 ±30.35
Ruptured into ventricles, n(%)	962 (29.6%)
Craniotomy, n(%)	81 (2.7%)
Withdrawal of support, n(%)	404 (12.4%)

SD= standard deviation, GCS=Glasgow Coma Score.

The in-hospital mortality rate was 12.0% (410 cases) while the 1-year overall mortality rate was 26.1%. The favorable prognosis rate (mRS≤2) was 50.9%, 50.9%, 52.9% and 54.0% at discharge and in 3, 6 and 12 months post ICH onset, respectively. The rate increased merely by 3.1% within 1 year period. However, the patients with a mRS of 0 increased by 300 cases (76.7%) during the same period while the patients with a mRS score of 1 or 2 decreased by 198 cases (15.7%). (Table 2)

**Table 2:** Modified Rankin Scale at different time points.

mRS	Discharge	3-month	6-month	12-month
0	391(12.0%)	566(17.4%)	596(18.3%)	691(21.2%)
1	1002(30.8%)	742(22.8%)	753(23.1%)	743(22.8%)
2	263(8.1%)	348(10.7%)	374(11.5%)	324(10.0%)
3	225(6.9%)	346(10.6%)	312(9.6%)	318(9.8%)
4	646(19.8%)	416(12.8%)	343(10.5%)	238(7.3%)
5	318(9.8%)	187(5.7%)	145(4.5)	92(2.8%)
6	410(12.6%)	650(20.0%)	732(22.5%)	849(26.1%)

mRS = modified Rankin ScalemRS: modified Rankin Scale

**Figure 2:**
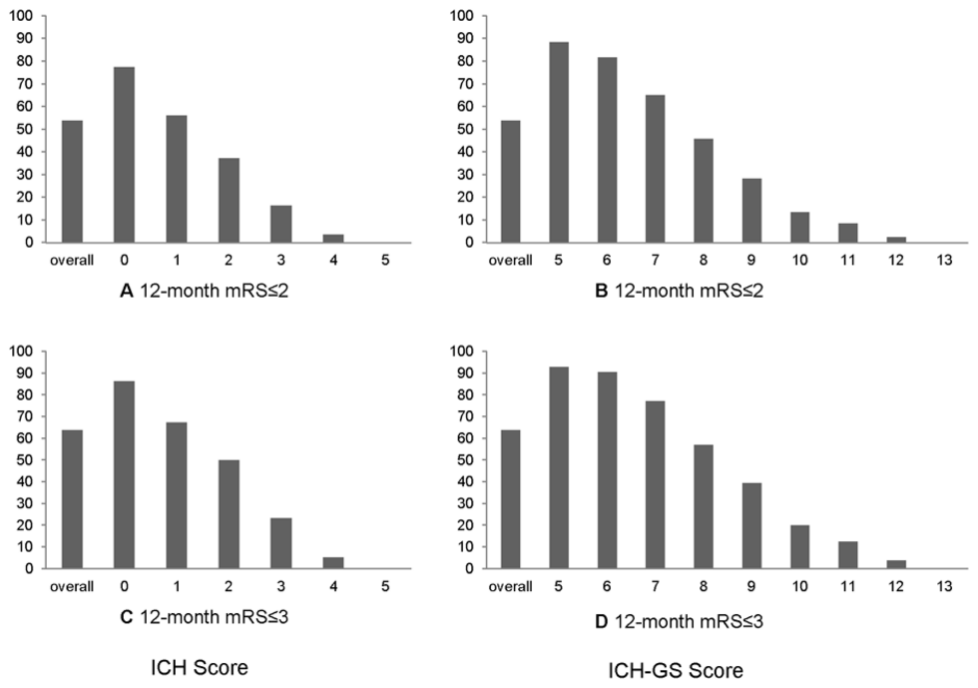
Favorable outcome based on various dichotomized mRScutoff points at 12-month follow-up. mRS = modified Rankin Scale, ICH= intracerebral hemorrhage, ICH-GS= intracerebral hemorrhage grading scale.

As shown in Figure 2, the favorable prognosis rate at discharge and 12 months after disease onset decreased gradually when the ICH score or ICH-GS score increased. The trend was similar when the cutoff point of favorable prognosis was set at either mRS=2 or mRS=3. 

AUC values were used to estimate the accuracy of ICH score and ICH-GS scorein predicting favorable prognosis. When mRS≤2 was selected as the cutoff point of favorable prognosis, the AUC values of ICH score were 0.72, 0.76, 0.76 and 0.75 at discharge, 3-, 6- and 12-months follow-ups, respectively; the AUC values of ICH-GS score at same time points were 0.71, 0.77, 0.78 and 0.78, respectively (Table 3). The ICH-GS score was clearly superior over the ICH score in predicting favorable prognosis at 6- and 12-month follow-ups (P=0.0003 and P<0.0001, respectively). 

**Table 3:** AUC of ICH score and ICH-GS score at different time points and mRS cutoff point.

		AUC for ICH score	AUC for ICH-GSscore	*P*
Hospital discharge	mRS≤2	0.72(0.70-0.74)	0.71(0.69-0.73)	0.0770
	mRS≤3	0.75(0.73-0.76)	0.74(0.72-0.76)	0.2187
3-month	mRS≤2	0.76(0.74-0.77)	0.77(0.76-0.79)	0.0057
	mRS≤3	0.79(0.77-0.80)	0.81(0.80-0.83)	<0.001
6-month	mRS≤2	0.76(0.75-0.78)	0.78(0.77-0.80)	0.0003
	mRS≤3	0.79(0.77-0.80)	0.81(0.80-0.83)	<0.0001
12-month	mRS≤2	0.75(0.73-0.77)	0.78(0.76-0.79)	<0.0001
	mRS≤3	0.77(0.76-0.79)	0.80(0.79-0.82)	<0.0001
1-year improvement of mRS from discharge		0.60(0.58-0.62)	0.64(0.62-0.66)	<0.0001

AUC= area under the curve, ICH= intracerebral hemorrhage, ICH-GS = intracerebral hemorrhage grading scale, mRS = modified Rankin Scale.

The correlation between the improvement of mRS and the ICH score or ICH-GS score was also analyzed. The AUC values of ICH score and ICH-GS score in predicting 1-year improvement of functional outcome were 0.60 and 0.64, respectively. The accuracy of the ICH-GS score was significantly better than that of the ICH score (P<0.0001, Table 3).

## Discussion

The ICH score is the most commonly used clinical grading scale in predicting the30-day mortality as well as the short-term and long-term functional outcome of ICH[17–23]. Currently, it has been widely recognized by the academic community as a useful prognostic evaluation scale[6,24]. Many revisions of the ICH score have been developed by investigators worldwide to try to improve the accuracy of the model. Among them, ICH-GS received more attention for its better accuracy in predicting the in-hospital and 30-day mortality as well as the 30-day favorable prognosis in patients with primary ICH[6].The ICH score and ICH-GS score are easy to use and can be steadily obtained during the patient’s first visit at most modern medical facilities[6]. Should they be validated by large-scale clinical studies, they could be of great values in prognosis estimate and could be used as important tools to compare the effects of different treatment modalities on the prognosis of ICH. However, there was no report about the predictive value of the two scores in China. 

CNSR is a nationwide and prospective registry of stroke patients in China. The registry collected comprehensive information during the patient’s hospital stay and the follow-up visits within 12 months post-onset from stroke patients across mainland China (including Hong Kong SAR). The registry is one of the best stroke databases currently available in China in terms of scale, complexity as well as demographic and geographic coverage. Our study took advantage of the CNSR database and was the first clinic trial to try to address the efficacy of the ICH score and ICH-GS score in predicting the short-term and long-term outcome of ICH in a large group of ICH patients in China.

This prospective study included a total of 3,255 ICH patients. There was a clear trend that the favorable prognosis rate decreased gradually with the increase of the ICH score and the ICH-GS score. The results were similar no matter mRS=2 or mRS=3 was selected as the cutoff point for favorable prognosis. This finding indicated that both the ICH score and the ICH-GS score were effective predictors for the functional outcome in Chinese ICH patients.

Our study also revealed that both the ICH score and the ICH-GS scores were able to accurately predict the favorable outcome of ICH patients at discharge and during all the follow-up visits. The trend of AUC values over time was similar when either mRS = 2 or mRS = 3 was used as the cutoff point of favorable outcome of ICH. However, the accuracy of the prediction for both the ICH score and the ICH-GS score with a cutoff point at 3 did show a slight edge over that with a cutoff point at 2, especially for the ICH-GS score and at 3-, 6- and 12-month follow-ups. Should ICH score or ICH-GS score be used for ICH prognosis prediction, mRS = 3 might be the better choice of cutoff point to compare the efficacy of the two models.

Ruiz-Sandovalet al reported that the ICH-GS score was clearly superior to the ICH score in predicting the in-hospital and 30-day mortality. Furthermore the ICH-GS score was also able to predict 30-day favorable outcome more accurately than the ICH score[25]. In our study, although the AUC values of the ICH score and ICH-GS score in predicting the favorable outcome of ICH were almost equal at discharge and 3-month follow-up, the accuracy of ICH-GS score at 6- and 12-month follow-ups presented a slight advantage over the ICH score. Our results were well in line with the finding in the other study. The advantage of the ICH-GS score over the ICH score might be the result of the refined ICH-GS scale method. The ICH-GS model uses an more detailed criteria to calculate the score. For example, to address the difference in compliance between different intracranial compartments, the ICH-GS model uses different cut off points to score the volume of hematoma from different locations (supratentorial or infratentorial). The cutoff points of age and GCS in ICH-GS scale model were also redefined to more accurately represent the pathophysiological change of aging and the impaired neurologic functions caused by ICH. Compared to ICH score, the ICH-GS scale model has a better stratification of various risk factors in ICH patients; hence it might be able to predict the outcome more accurately

We also observed that a significant portion of patients showed improvement in functional outcome in this study. Although 14.1% of the patients who were discharged from the hospital alive dead within 1 year, the number of patients with a mRS score of 0 also increased 77% (391 at discharge vs.691 at the 1-year follow-up).This polarizing trend in functional outcome warranted further studies. 

The preliminary results of our study suggested that both the ICH score and the ICH-GS score were effective in predicting the functional outcome of the Chinese ICH patients, but it is necessary to carry out more studies in the Chinese population to further confirm our findings.

Nevertheless some limitations in this study should be noted. First, up to 947 patients did not complete the 12-month follow-up and the rate was relatively high. Second, the evaluation of the functional outcome was conducted via phone during the follow-ups. The lack of on-site supervision by medical professionals might have some negative impact on the accuracy of the mRS score because some of the interviewees might not be able to answer the questions accurately. Finally, Chinese people were reluctant to withdraw the life support for their family member due to some cultural and religious reasons, even though the patients were confirmed at the permanent vegetative status. Therefore, the rate of withdrawal of life support was only 12.4% in our study and was much lower than the average rate reported by other studies. This could certainly have affected the results of our study.

## Conclusion

Both the ICH score and the ICH-GS score were able to accurately predict the short-term and long-term favorable functional outcome in Chinese ICH patients. Furthermore, the ICH-GS score was superior over the ICH score in predicting the 6-month and 12-month functional outcome.

## Supporting Information

Table S1 **Contents of ICH score and ICH GS score.**
(DOCX)
