# Supplementary material for: Prognostic Value of ICH Score and ICH-GS Score in Chinese Intracerebral Hemorrhage Patients: Analysis From the China National Stroke Registry (CNSR)
Source: PLoS One. 2013 Oct 16;8(10):e77421. doi: 10.1371/journal.pone.0077421 (PMC3797805; doi:10.1371/journal.pone.0077421)
Supplement: Table S1 — Contents of ICH score and ICH GS score. (DOCX) [file pone.0077421.s001.docx]

Table S1. Contens of ICH score and ICH GS score

| ICH score | | ICH GS score | |
| --- | --- | --- | --- |
| Items | Score | Items | Score |
| Age(years) |  | Age(years) |  |
| <80 | 0 | <45 | 1 |
| ≥80 | 1 | 45-64 | 2 |
|  |  | ≥65 | 3 |
| GCS score on admission |  | GCS score on admission |  |
| 13-15 | 0 | 13-15 | 1 |
| 9-12 | 1 | 9-12 | 2 |
| 3-8 | 2 | 3-8 | 3 |
| ICH site |  | ICH site |  |
| Supratentorial | 0 | Supratentorial | 1 |
| Infratentorial | 1 | Infratentorial | 2 |
| ICH volume |  | ICH volume |  |
| <30ml | 0 | Supratentorial |  |
| ≥30ml | 1 | <40ml | 1 |
|  |  | 40-70ml | 2 |
|  |  | >70ml | 3 |
|  |  | Infratentorial |  |
|  |  | <10ml | 1 |
|  |  | 10-20ml | 2 |
|  |  | >20ml | 3 |
| Ruptured into ventricle |  | Ruptured into ventricle |  |
| No | 0 | No | 0 |
| Yes | 1 | Yes | 1 |
